# Some *q*-supercongruences modulo the square and cube of a cyclotomic polynomial

**DOI:** 10.1007/s13398-021-01070-y

**Published:** 2021-05-31

**Authors:** Victor J. W. Guo, Michael J. Schlosser

**Affiliations:** 1 School of Mathematics and Statistics, Huaiyin Normal University, Huai’an, 223300 Jiangsu People’s Republic of China; 2 Fakultät für Mathematik, Universität Wien, Oskar-Morgenstern-Platz 1, 1090 Vienna, Austria

**Keywords:** Basic hypergeometric series, Supercongruences, *q*-congruences, Cyclotomic polynomial, Andrews’ transformation, Gasper’s summation, Primary 33D15, Secondary 11A07, 11B65

## Abstract

Two *q*-supercongruences of truncated basic hypergeometric series containing two free parameters are established by employing specific identities for basic hypergeometric series. The results partly extend two *q*-supercongruences that were earlier conjectured by the same authors and involve *q*-supercongruences modulo the square and the cube of a cyclotomic polynomial. One of the newly proved *q*-supercongruences is even conjectured to hold modulo the fourth power of a cyclotomic polynomial.

## Introduction

In 1914, Ramanujan [25] listed a number of representations of $1/\pi$, including

1.1 $$\begin{aligned} \sum _{k=0}^{\infty }(6k+1)\frac{\left( \frac{1}{2}\right) _k^3}{k!^3 4^k} =\frac{4}{\pi }, \end{aligned}$$

where $(a)_n=a(a+1)\cdots (a+n-1)$ denotes the Pochhammer symbol. Ramanujan’s formulas gained unprecedented popularity in the 1980’s when they were discovered to provide fast algorithms for calculating decimal digits of $\pi$. See, for instance, the monograph [2] by the Borwein brothers.

In 1997, Van Hamme [29] conjectured 13 intriguing *p*-adic analogues of Ramanujan-type formulas, such as

1.2 $$\begin{aligned} \sum _{k=0}^{(p-1)/2}(6k+1)\frac{(\frac{1}{2})_k^3}{k!^3 4^k}&\equiv p(-1)^{(p-1)/2}\pmod {p^4}, \end{aligned}$$

where $p>3$ is a prime. Van Hamme himself supplied proofs for three of them. Supercongruences like (1.2) are called Ramanujan-type supercongruences (see [33]). The proof of the supercongruence (1.2) was first given by Long [22]. As of today, all of Van Hamme’s 13 supercongruences have been confirmed by various techniques (see [24, 28]).

In recent years, *q*-congruences and *q*-supercongruences have been established by different authors (see, for example, [5–13, 15–21, 23, 27, 30–32, 34]). In particular, the present authors [9] proved that, for any odd integer $d\geqslant 5$,

1.3 $$\begin{aligned} \sum _{k=0}^{n-1}[2dk+1]\frac{(q;q^d)_k^d}{(q^d;q^d)_k^d}q^{d(d-3)k/2} \equiv {\left\{ \begin{array}{ll} 0\pmod {\Phi _n(q)^2}, &{}\text {if } n\equiv -1\pmod {d}, \\ 0\pmod {\Phi _n(q)^3}, &{}\text {if } n\equiv -1/2\pmod {d}. \end{array}\right. } \end{aligned}$$

Here and in what follows, we adopt the standard *q*-notation: $[n]=1+q+\cdots +q^{n-1}$ is the *q-integer*; $(a;q)_n=(1-a)(1-aq)\cdots (1-aq^{n-1})$ is the *q-shifted factorial*, with the compact notation $(a_1,a_2,\ldots ,a_m;q)_n=(a_1;q)_n (a_2;q)_n\cdots (a_m;q)_n$ used for their products; and $\Phi _n(q)$ denotes the *n*-th *cyclotomic polynomial* in *q*, which may be defined as

$$\begin{aligned} \Phi _n(q)=\prod _{\begin{array}{c} 1\leqslant k\leqslant n\\ \gcd (k,n)=1 \end{array}}(q-\zeta ^k), \end{aligned}$$

where $\zeta$ is an *n*-th primitive root of unity.

We should point out that the *q*-congruence (1.3) does not hold for $d=3$. The present authors [9] also established the following companion of (1.3): for any odd integer $d\geqslant 3$ and integer $n>1$,

1.4 $$\begin{aligned} \sum _{k=0}^{n-1}[2dk-1]\frac{(q^{-1};q^d)_k^d}{(q^d;q^d)_k^d} q^{d(d-1)k/2} \equiv {\left\{ \begin{array}{ll} 0\pmod {\Phi _n(q)^2}, &{}\text {if } n\equiv 1\pmod {d}, \\ 0\pmod {\Phi _n(q)^3}, &{}\text {if }n\equiv 1/2\pmod {d}. \end{array}\right. } \end{aligned}$$

They also proposed the following conjectures [9, Conjectures 1 and 2], which are generalizations of (1.3) and (1.4).

### Conjecture 1

Let $d\geqslant 5$ be an odd integer. Then

$$\begin{aligned} \sum _{k=0}^{n-1}[2dk+1]\frac{(q;q^d)_k^d}{(q^d;q^d)_k^d}q^{d(d-3)k/2} \equiv {\left\{ \begin{array}{ll} 0\pmod {\Phi _n(q)^3}, &{}\text {if } n\equiv -1\pmod {d}, \\ 0\pmod {\Phi _n(q)^4}, &{}\text {if } n\equiv -1/2\pmod {d}. \end{array}\right. } \end{aligned}$$

### Conjecture 2

Let $d\geqslant 5$ be an odd integer and let $n>1$. Then

$$\begin{aligned} \sum _{k=0}^{n-1}[2dk-1]\frac{(q^{-1};q^d)_k^d}{(q^d;q^d)_k^d}q^{d(d-1)k/2} \equiv {\left\{ \begin{array}{ll} 0\pmod {\Phi _n(q)^3}, &{}\text {if } n\equiv 1\pmod {d}, \\ 0\pmod {\Phi _n(q)^4}, &{}\text {if } n\equiv 1/2\pmod {d}. \end{array}\right. } \end{aligned}$$

*q*-Supercongruences such as those above (modulo a third and even fourth power of a cyclotomic polynomial) are rather special. In fact, concrete results for truncated basic hypergeometric sums being congruent to 0 modulo a high power of a cyclotomic polynomial are very rare. See [8, 10–12, 14, 18] for recent papers featuring such results. The main goal of this paper is to add two complete two-parameter families of *q*-supercongruences to the list of such *q*-supercongruences (see Theorems 1 and 2).

We shall prove that the respective first cases of Conjectures 1 and 2 are true by establishing the following more general result.

### Theorem 1

Let *d* and *r* be odd integers satisfying $d\geqslant 3$, $r\leqslant d-4$ (in particular, *r* may be negative) and $\gcd (d,r)=1$. Let *n* be an integer such that $n\geqslant d-r$ and $n\equiv -r\pmod {d}$. Then

1.5 $$\begin{aligned} \sum _{k=0}^{M}[2dk+r]\frac{(q^r;q^d)_k^d}{(q^d;q^d)_k^d}q^{d(d-r-2)k/2} \equiv 0 \pmod {[n]\Phi _{n}(q)^2}, \end{aligned}$$

where $$M=(dn-n-r)/d$$ or $$n-1$$.

We shall also prove the following *q*-supercongruences.

### Theorem 2

Let *d* and *r* be odd integers satisfying $d\geqslant 3$, $r\leqslant d-4$ (in particular, *r* may be negative) and $\gcd (d,r)=1$. Let *n* be an integer such that $n\geqslant (d-r)/2$ and $n\equiv -r/2\pmod {d}$. Then

1.6 $$\begin{aligned} \sum _{k=0}^{M}[2dk+r]\frac{(q^r;q^d)_k^d}{(q^d;q^d)_k^d}q^{d(d-r-2)k/2} \equiv 0 \pmod {[n]\Phi _{n}(q)}, \end{aligned}$$

where $$M=(dn-2n-r)/d$$ or $$n-1$$.

The following generalization of the respective second cases of Conjectures 1 and 2 should be true.

### Conjecture 3

The *q*-supercongruence (1.6) holds modulo $[n]\Phi _{n}(q)^3$ for $d\geqslant 5$.

We shall prove Theorems 1 and 2 in Sections 2 and 3, respectively, by making use of Andrews’ multiseries extension (2.2) of the Watson transformation [1, Theorem 4], along with Gasper’s very-well-poised Karlsson–Minton type summation [3, Eq. (5.13)]. It should be pointed out that Andrews’ transformation plays an important part in combinatorics and number theory (see [7] and the introduction of [12] for more such examples).

## Proof of Theorem 1

We need a simple *q*-congruence modulo $\Phi _n(q)^2$, which was already used in [10, 12].

### Lemma 1

Let $\alpha$, *r* be integers and *n* a positive integer. Then

2.1 $$\begin{aligned} (q^{r-\alpha n},q^{r+\alpha n};q^d)_k \equiv (q^r;q^d)_k^2 \pmod {\Phi _n(q)^2}. \end{aligned}$$

We will further utilize a powerful transformation formula due to Andrews [1, Theorem 4], which may be stated as follows:

2.2 $$\begin{aligned}&\sum _{k\geqslant 0}\frac{(a,q\sqrt{a},-q\sqrt{a},b_1,c_1,\dots ,b_m,c_m,q^{-N};q)_k}{(q,\sqrt{a},-\sqrt{a},aq/b_1,aq/c_1,\dots ,aq/b_m,aq/c_m,aq^{N+1};q)_k} \left( \frac{a^mq^{m+N}}{b_1c_1\cdots b_mc_m}\right) ^k \nonumber \\&\quad =\frac{(aq,aq/b_mc_m;q)_N}{(aq/b_m,aq/c_m;q)_N} \sum _{j_1,\dots ,j_{m-1}\geqslant 0} \frac{(aq/b_1c_1;q)_{j_1}\cdots (aq/b_{m-1}c_{m-1};q)_{j_{m-1}}}{(q;q)_{j_1}\cdots (q;q)_{j_{m-1}}} \nonumber \\&\qquad \quad \times \frac{(b_2,c_2;q)_{j_1}\dots (b_m,c_m;q)_{j_1+\dots +j_{m-1}}}{(aq/b_1,aq/c_1;q)_{j_1} \dots (aq/b_{m-1},aq/c_{m-1};q)_{j_1+\dots +j_{m-1}}} \nonumber \\&\qquad \quad \times \frac{(q^{-N};q)_{j_1+\dots +j_{m-1}}}{(b_mc_mq^{-N}/a;q)_{j_1+\dots +j_{m-1}}} \frac{(aq)^{j_{m-2}+\dots +(m-2)j_1} q^{j_1+\dots +j_{m-1}}}{(b_2c_2)^{j_1}\cdots (b_{m-1}c_{m-1})^{j_1+\dots +j_{m-2}}}. \end{aligned}$$

This transformation is a multiseries generalization of Watson’s $_8\phi _7$ transformation formula (listed in [4, Appendix (III.18)]; cf. [4, Chapter 1] for the notation of a basic hypergeometric $_r\phi _s$ series we are using),

2.3 $$\begin{aligned} _{8}\phi _{7}\!&\left[ \begin{array}{cccccccc} a,&{} qa^{\frac{1}{2}},&{} -qa^{\frac{1}{2}}, &{} b, &{} c, &{} d, &{} e, &{} q^{-n} \\ &{} a^{\frac{1}{2}}, &{} -a^{\frac{1}{2}}, &{} aq/b, &{} aq/c, &{} aq/d, &{} aq/e, &{} aq^{n+1} \end{array};q,\, \frac{a^2q^{n+2}}{bcde} \right] \nonumber \\&\quad =\frac{(aq, aq/de;q)_n}{(aq/d, aq/e;q)_n} \,{}_{4}\phi _{3}\!\left[ \begin{array}{c} aq/bc,\ d,\ e,\ q^{-n} \\ aq/b,\, aq/c,\, deq^{-n}/a \end{array};q,\, q \right] , \end{aligned}$$

to which it reduces for $m=2$.

Next, we require a very-well-poised Karlsson–Minton type summation due to Gasper [3, Eq. (5.13)] (see also [4, Ex. 2.33 (i)]):

2.4 $$\begin{aligned} \sum _{k=0}^\infty&\frac{(a,q\sqrt{a},-q\sqrt{a},b,a/b,d,e_1,aq^{n_1+1}/e_1,\dots , e_m,aq^{n_m+1}/e_m;q)_k}{(q,\sqrt{a},-\sqrt{a},aq/b,bq,aq/d,aq/e_1,e_1q^{-n_1}, \dots ,aq/e_m,e_mq^{-n_m};q)_k}\left( \frac{q^{1-\nu }}{d}\right) ^k \nonumber \\&\quad =\frac{(q,aq,aq/bd,bq/d;q)_\infty }{(bq,aq/b,aq/d,q/d;q)_\infty } \prod _{j=1}^m\frac{(aq/be_j,bq/e_j;q)_{n_j}}{(aq/e_j,q/e_j;q)_{n_j}}, \end{aligned}$$

where $n_1,\dots ,n_m$ are non-negative integers, $\nu =n_1+\cdots +n_m$, and the convergence condition $|q^{1-\nu }/d|<1$ is required if the series does not terminate. We point out that an elliptic extension of the terminating $d=q^{-\nu }$ case of (2.4) can be found in [26, Eq. (1.7)].

In particular, we note that for $d=bq$ the right-hand side of (2.4) vanishes. Putting in addition $b=q^{-N}$ we get the following terminating summation formula:

2.5 $$\begin{aligned} \sum _{k=0}^N\frac{(a,q\sqrt{a},-q\sqrt{a},e_1,aq^{n_1+1}/e_1,\dots , e_m,aq^{n_m+1}/e_m,q^{-N};q)_k}{(q,\sqrt{a},-\sqrt{a},aq/e_1,e_1q^{-n_1}, \dots ,aq/e_m,e_mq^{-n_m},aq^{N+1};q)_k}q^{(N-\nu )k}=0, \end{aligned}$$

which is valid for $N>\nu =n_1+\cdots +n_m$.

A suitable combination of (2.2) and (2.5) yields the following multi-series summation formula, derived in [12, Lemma 2] (whose proof we nevertheless give here, to make the paper self-contained):

### Lemma 2

Let $m\geqslant 2$. Let *q*, *a* and $e_1,\dots ,e_{m+1}$ be arbitrary parameters with $e_{m+1}=e_1$, and let $n_1,\dots ,n_m$ and *N* be non-negative integers such that $N>n_1+\cdots +n_m$. Then

2.6 $$\begin{aligned} 0=&\sum _{j_1,\dots ,j_{m-1}\geqslant 0} \frac{(e_1q^{-n_1}/e_2;q)_{j_1}\cdots (e_{m-1}q^{-n_{m-1}}/e_m;q)_{j_{m-1}}}{(q;q)_{j_1}\cdots (q;q)_{j_{m-1}}} \nonumber \\&\times \frac{(aq^{n_2+1}/e_2,e_3;q)_{j_1}\dots (aq^{n_m+1}/e_m,e_{m+1};q)_{j_1+\dots +j_{m-1}}}{(e_1q^{-n_1},aq/e_2;q)_{j_1} \dots (e_{m-1}q^{-n_{m-1}},aq/e_m;q)_{j_1+\dots +j_{m-1}}} \nonumber \\&\times \frac{(q^{-N};q)_{j_1+\dots +j_{m-1}}}{(e_1q^{n_m-N+1}/e_m;q)_{j_1+\dots +j_{m-1}}} \frac{(aq)^{j_{m-2}+\dots +(m-2)j_1} q^{j_1+\dots +j_{m-1}}}{(aq^{n_2+1}e_3/e_2)^{j_1}\cdots (aq^{n_{m-1}+1}e_m/e_{m-1})^{j_1+\dots +j_{m-2}}}. \end{aligned}$$

### Proof

By specializing the parameters in the multi-sum transformation (2.2) by $b_i\mapsto aq^{n_i+1}/e_i$, $c_i\mapsto e_{i+1}$, for $1\le i\le m$ (where $e_{m+1}=e_1$), and dividing both sides of the identity by the prefactor of the multi-sum, we obtain that the series on the right-hand side of (2.6) equals

$$\begin{aligned} \frac{(e_mq^{-n_m},aq/e_1;q)_N}{(aq,e_mq^{-n_m}/e_1;q)_N} \times \sum _{k=0}^N\frac{(a,q\sqrt{a},-q\sqrt{a},e_1,aq^{n_1+1}/e_1,\dots , e_m,aq^{n_m+1}/e_m,q^{-N};q)_k}{(q,\sqrt{a},-\sqrt{a},aq/e_1,e_1q^{-n_1}, \dots ,aq/e_m,e_mq^{-n_m},aq^{N+1};q)_k}q^{(N-\nu )k}, \end{aligned}$$

with $\nu =n_1+\cdots +n_m$. Now the last sum vanishes by the special case of Gasper’s summation stated in (2.5). $\square$

Using [11, Lemma 2.1], we can prove the following result which is similar to [11, Lemma 2.2].

### Lemma 3

Let *d*, *n* be positive integers with $\gcd (d,n)=1$. Let *r* be an integer. Then

$$\begin{aligned} \sum _{k=0}^{m} [2dk+r]\frac{(q^r;q^d)_k^d}{(q^d;q^d)_k^d}q^{d(d-r-2)k/2} \equiv 0\pmod {[n]}, \\ \sum _{k=0}^{n-1} [2dk+r]\frac{(q^r;q^d)_k^d}{(q^d;q^d)_k^d}q^{d(d-r-2)k/2} \equiv 0\pmod {[n]}, \end{aligned}$$

where $$0\leqslant m\leqslant n-1$$ and $$dm\equiv -r\pmod {n}$$.

We have collected enough ingredients which enables us to prove Theorem 1.

### Proof of Theorem 1

The *q*-congruence (1.5) modulo [*n*] follows from Lemma 3 immediately. In what follows, we shall prove the modulus $\Phi _n(q)^3$ case of (1.5).

For $M=(dn-n-r)/d$, the left-hand side of (1.5) can be written as the following multiple of a terminating $_{d+5}\phi _{d+4}$ series:

$$\begin{aligned} {[}r] \sum _{k=0}^{(dn-n-r)/d}\frac{(q^r,q^{d+r/2},-q^{d+r/2}, q^r,\ldots ,q^r,q^{(d+r)/2},q^{d+(d-1)n},q^{r-(d-1)n};q^d)_k}{(q^d,q^{r/2},-q^{r/2},q^d,\ldots ,q^d,q^{(d+r)/2},q^{r-(d-1)n},q^{d+(d-1)n};q^d)_k}q^{d(d-r-2)k/2}. \end{aligned}$$

Here, the $q^r,\ldots ,q^r$ in the numerator means $d-1$ instances of $q^r$, and similarly, the $q^d,\ldots ,q^d$ in the denominator means $d-1$ instances of $q^d$. By Andrews’ transformation (2.2), we may rewrite the above expression as

2.7 $$\begin{aligned}&{[}r]\frac{(q^{d+r},q^{(r-d)/2-(d-1)n};q^d)_{(dn-n-r)/d}}{(q^{(d+r)/2},q^{r-(d-1)n};q^d)_{(dn-n-r)/d}} \sum _{j_1,\dots ,j_{m-1}\geqslant 0} \frac{(q^{d-r};q^d)_{j_1}\cdots (q^{d-r};q^d)_{j_{m-1}}}{(q^d;q^d)_{j_1}\cdots (q^d;q^d)_{j_{m-1}}} \nonumber \\&\quad \times \frac{(q^r,q^r;q^d)_{j_1}\dots (q^r,q^r;q^d)_{j_1+\dots +j_{m-2}} (q^{(d+r)/2},q^{d+(d-1)n};q^d)_{j_1+\dots +j_{m-1}}}{(q^d,q^d;q^d)_{j_1} \dots (q^d,q^d;q^d)_{j_1+\dots +j_{m-1}}} \nonumber \\&\quad \times \frac{(q^{r-(d-1)n};q^d)_{j_1+\dots +j_{m-1}}}{(q^{(3d+r)/2};q^d)_{j_1+\dots +j_{m-1}}} q^{(d-r)(j_{m-2}+\dots +(m-2)j_1)+d(j_1+\dots +j_{m-1})} , \end{aligned}$$

where $$m=(d+1)/2$$.

It is easy to see that the *q*-shifted factorial $(q^{d+r};q^d)_{(dn-n-r)/d}$ contains the factor $1-q^{(d-1)n}$ which is a multiple of $1-q^n$. Moreover, since none of $(r-d)/2$, $(d+r)/2$ and $(d+r)/2+dn-n-r-d$ are multiples of *n*, the *q*-shifted factorials

$$\begin{aligned} (q^{(r-d)/2-(d-1)n};q^d)_{(dn-n-r)/d} \quad \text {and} \quad (q^{(d+r)/2};q^d)_{(dn-n-r)/d} \end{aligned}$$

have the same number (0 or 1) of factors of the form $1-q^{\alpha n}$ ($\alpha \in \mathbb {Z}$). Besides, the *q*-shifted factorial $(q^{r-(d-1)n};q^d)_{(dn-n-r)/d}$ is relatively prime to $\Phi _n(q)$. Thus we conclude that the fraction before the multi-sum in (2.7) is congruent to 0 modulo $\Phi _n(q)$.

Note that the non-zero terms in the multi-summation in (2.7) are those indexed by $(j_1,\ldots ,j_{m-1})$ that satisfy the inequality $j_1+\dots +j_{m-1}\leqslant (dn-n-r)/d$ because the factor $(q^{r-(d-1)n};q^d)_{j_1+\dots +j_{m-1}}$ appears in the numerator. None of the factors appearing in the denominator of the multi-sum of (2.7) contain a factor of the form $1-q^{\alpha n}$ (and are therefore relatively prime to $\Phi _n(q)$), except for $(q^{(3d+r)/2};q^d)_{j_1+\dots +j_{m-1}}$ when

$$\begin{aligned} (dn-d-n-r)/(2d)\leqslant j_1+\dots +j_{m-1}\leqslant (dn-n-r)/d. \end{aligned}$$

Since

$$\begin{aligned} \frac{(q^{(d+r)/2};q^d)_{j_1+\dots +j_{m-1}}}{(q^{(3d+r)/2};q^d)_{j_1+\dots +j_{m-1}}} =\frac{1-q^{(d+r)/2}}{1-q^{(d+r)/2+(j_1+\dots +j_{m-1})d}}, \end{aligned}$$

the denominator of the above fraction contains a factor of the form $1-q^{\alpha n}$ if and only if $j_1+\dots +j_{m-1}=(dn-d-n-r)/(2d)$ (in this case, the denominator contains the factor $1-q^{(d-1)n/2}$). Writing $n=ad-r$ (with $a\geqslant 1$), we have $j_1+\dots +j_{m-1}=a(d-1)/2-(r+1)/2$. Noticing that $m-1=(d-1)/2$ and $r\leqslant d-4$, there must exist an *i* such that $j_i\geqslant a$. Then $(q^{d-r};q^d)_{j_i}$ has the factor $1-q^{d-r+d(a-1)}=1-q^n$ which is divisible by $\Phi _n(q)$. Hence the denominator of the reduced form of the multi-sum in (2.7) is relatively prime to $\Phi _n(q)$. It remains to show that the multi-sum in (2.7), without the previous fraction, is congruent to 0 modulo $\Phi _n(q)^2$.

By repeated applications of Lemma 1, the multi-sum in (2.7) (without the previous fraction), modulo $\Phi _n(q)^2$, is congruent to

$$\begin{aligned} \sum _{j_1,\dots ,j_{m-1}\geqslant 0}&q^{(d-r)(j_{m-2}+\dots +(m-2)j_1)+d(j_1+\dots +j_{m-1})} \frac{(q^{d-r};q^d)_{j_1}\cdots (q^{d-r};q^d)_{j_{m-1}}}{(q^d;q^d)_{j_1}\cdots (q^d;q^d)_{j_{m-1}}} \nonumber \\&\quad \times \frac{(q^{r+(m+1)n},q^{r-(m+1)n};q^d)_{j_1}\dots (q^{r+(2m-2)n},q^{r-(2m-2)n};q^d)_{j_1+\dots +j_{m-2}} }{(q^{d-mn},q^{d+mn};q^d)_{j_1} \dots (q^{d-(2m-3)n},q^{d+(2m-3)n};q^d)_{j_1+\dots +j_{m-2}} } \nonumber \\&\quad \times \frac{(q^{d+(d-1)n},q^{(d+r)/2};q^d)_{j_1+\dots +j_{m-1}} (q^{r-(d-1)n};q^d)_{j_1+\dots +j_{m-1}}}{(q^{d-(2m-2)n},q^{d+(2m-2)n};q^d)_{j_1+\dots +j_{m-1}} (q^{(3d+r)/2};q^d)_{j_1+\dots +j_{m-1}}} , \end{aligned}$$

where $m=(d+1)/2$. However, this sum vanishes in light of the $m=(d+1)/2$, $q\mapsto q^d$, $a= q^r$, $e_1=q^{(d+r)/2}$, $e_m=q^{r-(2m-2)n}$, $e_i= q^{r-(m+i-2)n}$, $n_1=(dn-d+n+r)/(2d)$, $n_m=0$, $n_i= (n+r-d)/d$, $2\leqslant i\leqslant m-1$, $N=(dn-n-r)/d$ case of Lemma 2. (It is easy to verify that $N-n_1-\cdots -n_m=d(d-r-2)/2>0$.) This proves that (1.5) holds modulo $\Phi _n(q)^3$ for $M=(dn-n-r)/d$.

Since $(q^r;q^d)_k/(q^d;q^d)_k$ is congruent to 0 modulo $\Phi _n(q)$ for $(dn-n-r)/d<k\leqslant n-1$, we conclude that (1.5) also holds modulo $\Phi _n(q)^3$ for $M=n-1$. $\square$

## Proof of Theorem 2

We first give a simple lemma on a property of certain arithmetic progressions.

### Lemma 4

Let *d* and *r* be odd integers satisfying $d\geqslant 3$, $r\leqslant d-4$ and $\gcd (d,r)=1$. Let *n* be an integer such that $n\geqslant (d-r)/2$ and $n\equiv -r/2\pmod {d}$. Then there are no multiples of *n* in the arithmetic progression

3.1 $$\begin{aligned} \frac{d+r}{2},\ \frac{d+r}{2}+d,\ldots , \frac{d+r}{2}+dn-2n-r-d. \end{aligned}$$

### Proof

By the condition $\gcd (d,r)=1$, we have $\gcd ((d+r)/2,(d-r)/2)=1$. Suppose that

3.2 $$\begin{aligned} (d+r)/2+ad=bn \end{aligned}$$

for some integers *a* and *b* with $a\geqslant 0$. Then $(d+r)/2+ad> (r-d)/2\geqslant -n$ and so $b\geqslant 0$. Since $n\equiv (d-r)/2\pmod {d}$, we deduce from (3.2) that $b\equiv -1\pmod {d}$ and thereby $b\geqslant d-1$. But we have

$$\begin{aligned} \frac{d+r}{2}+dn-2n-r-d =dn-2n+\frac{d-r}{2}-d\leqslant (d-1)n-d, \end{aligned}$$

thus implying that no number in the arithmetic progression (3.1) is a multiple of *n*. $\square$

### Proof of Theorem 2

As before, the *q*-congruence (1.6) modulo [*n*] can be deduced from Lemma 3. It remains to prove the modulus $\Phi _n(q)^2$ case of (1.6).

For $M=(dn-2n-r)/d$, the left-hand side of (1.6) can be written as the following multiple of a terminating $_{d+5}\phi _{d+4}$ series (this time we changed the position of $q^{(d+r)/2}$):

$$\begin{aligned} {[}r]&\sum _{k=0}^{(dn-2n-r)/d}\frac{(q^r,q^{d+r/2},-q^{d+r/2},q^{(d+r)/2}, q^r,\ldots ,q^r,q^{d+(d-2)n},q^{r-(d-2)n};q^d)_k}{(q^d,q^{r/2},-q^{r/2},q^{(d+r)/2},q^d,\ldots ,q^d,q^{r-(d-2)n},q^{d+(d-2)n};q^d)_k}\\&\quad \times q^{d(d-r-2)k/2}. \end{aligned}$$

Here, the $q^r,\ldots ,q^r$ in the numerator stands for $d-1$ instances of $q^r$, and similarly, the $q^d,\ldots ,q^d$ in the denominator stands for $d-1$ instances of $q^d$. By Andrews’ transformation (2.2), we may rewrite the above expression as

3.3 $$\begin{aligned}&{[}r]\frac{(q^{d+r},q^{-(d-2)n};q^d)_{(dn-2n-r)/d}}{(q^d,q^{r-(d-2)n};q^d)_{(dn-2n-r)/d}} \sum _{j_1,\dots ,j_{m-1}\geqslant 0} \frac{(q^{(d-r)/2};q^d)_{j_1}(q^{d-r};q^d)_{j_2}\cdots (q^{d-r};q^d)_{j_{m-1}}}{(q^d;q^d)_{j_1}(q^d;q^d)_{j_2}\cdots (q^d;q^d)_{j_{m-1}}} \nonumber \\&\qquad \quad \times \frac{(q^r,q^r;q^d)_{j_1}\dots (q^r,q^r;q^d)_{j_1+\dots +j_{m-2}} (q^r,q^{d+(d-2)n};q^d)_{j_1+\dots +j_{m-1}}}{(q^{(d+r)/2},q^d;q^d)_{j_1}(q^d,q^d;q^d)_{j_1+j_2} \dots (q^d,q^d;q^d)_{j_1+\dots +j_{m-1}}} \nonumber \\&\qquad \quad \times \frac{(q^{r-(d-2)n};q^d)_{j_1+\dots +j_{m-1}}}{(q^{d+r};q^d)_{j_1+\dots +j_{m-1}}} q^{(d-r)(j_{m-2}+\dots +(m-2)j_1)+d(j_1+\dots +j_{m-1})} , \end{aligned}$$

where $$m=(d+1)/2$$.

It is easily seen that the *q*-shifted factorial $(q^{d+r};q^d)_{(dn-2n-r)/d}$ has the factor $1-q^{(d-2)n}$ which is a multiple of $1-q^n$. Clearly, the *q*-shifted factorial $(q^{-(d-2)n};q^d)_{(dn-2n-r)/d}$ has the factor $1-q^{-(d-1)n}$ (again being a multiple of $1-q^n$) since $(dn-2n-r)/d\geqslant 1$ holds according to the conditions $d\geqslant 3$, $r\leqslant d-4$, and $n\geqslant (d-r)/2$. This indicates that the *q*-factorial $(q^{d+r},q^{-(d-2)n};q^d)_{(dn-2n-r)/d}$ in the numerator of the fraction before the multi-sum in (3.3) is divisible by $\Phi _n(q)^2$. Further, it is not difficult to see that the *q*-factorial $(q^d,q^{r-(d-2)n};q^d)_{(dn-2n-r)/d}$ in the denominator is relatively prime to $\Phi _n(q)$.

Like the proof of Theorem 1, the non-zero terms in the multi-sum in (3.3) are those indexed by $(j_1,\ldots ,j_{m-1})$ satisfying the inequality $j_1+\dots +j_{m-1}\leqslant (dn-2n-r)/d$ because of the appearance of the factor $(q^{r-(d-2)n};q^d)_{j_1+\dots +j_{m-1}}$ in the numerator. By Lemma 4, the *q*-shifted factorial $(q^{(d+r)/2},q^d)_{j_1}$ in the denominator does not contain a factor of the form $1-q^{\alpha n}$ for $j_1\leqslant (dn-2n-r)/d$ (and are therefore relatively prime to $\Phi _n(q)$). In addition, none of the other factors appearing in the denominator of the multi-sum of (3.3) contain a factor of the form $1-q^{\alpha n}$, except for $(q^{d+r};q^d)_{j_1+\dots +j_{m-1}}$ when $j_1+\dots +j_{m-1}=(dn-2n-r)/d$ (in this case the denominator contains the factor $1-q^{(d-2)n}$).

Letting $n=ad+(d-r)/2$ (with $a\geqslant 0$), we get $j_1+\dots +j_{m-1}=a(d-2)+(d-r)/2-1$. If $j_1\geqslant a+1$, then $(q^{(d-r)/2};q^d)_{j_1}$ contains the factor $1-q^{(d-r)/2+ad}=1-q^n$. If $j_1\leqslant a$, then $j_2+\dots +j_{m-1}\geqslant a(d-3)+(d-r)/2-1$. Since $m-2=(d-3)/2$, $d\geqslant 3$, and $r\leqslant d-4$, there must be an *i* with $2\leqslant i\leqslant m-1$ and $j_i\geqslant 2a+1$. Then $(q^{d-r};q^d)_{j_i}$ contains the factor $1-q^{d-r+2ad}=1-q^{2n}$ which is a multiple of $\Phi _n(q)$. Therefore, the denominator of the reduced form of the multi-sum in (3.3) is relatively prime to $\Phi _n(q)$. This proves that (3.3) is congruent to 0 modulo $\Phi _n(q)^2$.

For $M=n-1$, since $(q^r;q^d)_k/(q^d;q^d)_k$ is congruent to 0 modulo $\Phi _n(q)$ for $(dn-2n-r)/d<k\leqslant n-1$, we conclude that (1.6) is also true modulo $\Phi _n(q)^2$ in this case. $\square$
